# Creative resources and affective modulation: a study conducted with students at the Institute of Arts and Crafts of Sfax

**DOI:** 10.1192/j.eurpsy.2026.11503

**Published:** 2026-07-31

**Authors:** N. Regaieg, H. Mezghani, F. Guermazi, R. Masmoudi, I. Feki, J. Masmoudi, I. Baati

**Affiliations:** Psychiatry A Department, Hedi Chaker University Hospital, Sfax, Tunisia

## Abstract

**Introduction:**

Various cognitive and environmental factors influence an individual’s creative abilities. However, the link between creative potential and certain affective traits remains largely unexplored, particularlyamong artistic populations.

**Objectives:**

The objective of this study is to investigate the links between different affective temperaments and creative skills in a population of students from the Institute of Arts and Crafts of Sfax.

**Methods:**

Our study was cross-sectional, descriptive and analytical, conducted between February 1^st^ to March 5^th^, 2025, among students at the Institute of Arts and Crafts in Sfax. Data were collected using a form that included sociodemographic and clinical characteristics, as well as two psychometric scales in their Arabic versions: Epstein Creativity Competencies Inventory for Individuals (ECCI-i) to assess creative skills and Tunisian TEMPERAMENT Scale (TEMPS) to measure affective temperaments.

**Results:**

A total of 60 students participated in the study, divided into 23 men and 37 women, with a sex ratio of 0.6. The median age was 20 years [19-22 years].

Regarding creative skills, 3.3% of students displayed weak creative skills, 23.3% average creative skills, and 73.3% high creative skills.

Furthermore, 76.7% of students reported that their creativity or artistic work strongly influenced their emotions. Additionally, 61.7% of participants reported feeling more creative during periods of sadness or euphoria.

Our study revealed that students with high creative skills had significantly higher scores on cyclothymic and hyperthymic temperament compared to those with average or low creative skills (p = 0.001). However, depressive, irritable, and anxious temperaments did not show a significant association with creative skills.

**Conclusions:**

These results support a strong link between creative skills and affective profiles characterized by instability, reactivity, and dynamism. A better understanding of these interactions could lead to improved support for these students in managing their emotions and optimizing their creative potential.

**Disclosure of Interest:**

None Declared

